# Comparative effectiveness of mRNA-1273 and BNT162b2 COVID-19 vaccines in immunocompromised individuals: a systematic review and meta-analysis using the GRADE framework

**DOI:** 10.3389/fimmu.2023.1204831

**Published:** 2023-09-12

**Authors:** Xuan Wang, Katrin Haeussler, Anne Spellman, Leslie E. Phillips, Allison Ramiller, Mary T. Bausch-Jurken, Pawana Sharma, Anna Krivelyova, Sonam Vats, Nicolas Van de Velde

**Affiliations:** ^1^ ICON plc, Stockholm, Sweden; ^2^ ICON plc, Munich, Germany; ^3^ Data Health Ltd, London, United Kingdom; ^4^ Data-Driven LLC, Seattle, WA, United States; ^5^ Moderna, Inc., Cambridge, MA, United States; ^6^ ICON plc, London, United Kingdom; ^7^ ICON plc, Bengaluru, India

**Keywords:** severe acute respiratory syndrome coronavirus 2, SARS-CoV-2, COVID-19, mRNA vaccine, mRNA-1273, BNT162b2, immunocompromised, effectiveness

## Abstract

**Introduction:**

Despite representing only 3% of the US population, immunocompromised (IC) individuals account for nearly half of the COVID-19 breakthrough hospitalizations. IC individuals generate a lower immune response after vaccination in general, and the US CDC recommended a third dose of either mRNA-1273 or BNT162b2 COVID-19 vaccines as part of their primary series. Influenza vaccine trials have shown that increasing dosage could improve effectiveness in IC populations. The objective of this systematic literature review and pairwise meta-analysis was to evaluate the clinical effectiveness of mRNA-1273 (50 or 100 mcg/dose) vs BNT162b2 (30 mcg/dose) in IC populations using the GRADE framework.

**Methods:**

The systematic literature search was conducted in the World Health Organization COVID-19 Research Database. Studies were included in the pairwise meta-analysis if they reported comparisons of mRNA-1273 and BNT162b2 in IC individuals ≥18 years of age; outcomes of interest were symptomatic, laboratory-confirmed SARS-CoV-2 infection, SARS-CoV-2 infection, severe SARS-CoV-2 infection, hospitalization due to COVID-19, and mortality due to COVID-19. Risk ratios (RR) were pooled across studies using random-effects meta-analysis models. Outcomes were also analyzed in subgroups of patients with cancer, autoimmune disease, and solid organ transplant. Risk of bias was assessed using the Newcastle-Ottawa Scale for observational studies. Evidence was evaluated using the GRADE framework.

**Results:**

Overall, 17 studies were included in the pairwise meta-analysis. Compared with BNT162b2, mRNA-1273 was associated with significantly reduced risk of SARS-CoV-2 infection (RR, 0.85 [95% CI, 0.75–0.97]; *P*=0.0151; *I^2 ^= *67.7%), severe SARS-CoV-2 infection (RR, 0.85 [95% CI, 0.77–0.93]; *P*=0.0009; *I^2 ^= *0%), COVID-19–associated hospitalization (RR, 0.88 [95% CI, 0.79–0.97]; *P*<0.0001; *I^2 ^= *0%), and COVID-19–associated mortality (RR, 0.63 [95% CI, 0.44–0.90]; *P*=0.0119; *I^2 ^= *0%) in IC populations. Results were consistent across subgroups. Because of sample size limitations, relative effectiveness of COVID-19 mRNA vaccines in IC populations cannot be studied in randomized trials. Based on nonrandomized studies, evidence certainty among comparisons was type 3 (low) and 4 (very low), reflecting potential biases in observational studies.

**Conclusion:**

This GRADE meta-analysis based on a large number of consistent observational studies showed that the mRNA-1273 COVID-19 vaccine is associated with improved clinical effectiveness in IC populations compared with BNT162b2.

## 1 Introduction

The global coronavirus disease 2019 (COVID-19) pandemic caused by severe acute respiratory syndrome coronavirus 2 (SARS-CoV-2) has resulted in 103 million reported infections and 1.1 million deaths to date in the United States (1). In response to the pandemic, mRNA-1273 (Spikevax^®^, Moderna, Inc., Cambridge, MA, USA) (2) and BNT162b2 (Comirnaty^®^, Pfizer/BioNTech, New York, NY, USA/Mainz, Germany) (3), each employing novel messenger ribonucleic acid (mRNA) technology, were developed and approved for the prevention of COVID-19 (4). Global phase 2/3 studies demonstrated that both mRNA vaccines given in a 2-dose series were highly efficacious at reducing symptomatic infections and hospitalizations in the immunocompetent population (5, 6).

Although immunocompromised (IC) individuals comprise only approximately 3% of the US population (7), they account for nearly half of the breakthrough COVID-19 hospitalizations (8). While there is a range of severity across conditions at the population level, adults considered immunodeficient had 2.68-fold greater adjusted odds of being hospitalized with COVID-19 compared with immunocompetent individuals due both to the underlying IC condition and therapies used for treatment (9, 10). In 1 study, use of immunosuppression in patients with autoimmune disease resulted in 1.35-fold (95% CI, 1.29–1.40) greater odds of developing life-threatening COVID-19 (11).

Despite being at increased risk of COVID-19–related morbidity and mortality (10, 12–14), IC individuals and patients receiving immunosuppressive medications were excluded from participating in pivotal trials of mRNA-1273 and BNT162b2 (5, 6). Real-world COVID-19 data indicate that vaccine immune responses are generally impaired in IC populations (9, 15–17) and that vaccine effectiveness, estimated as the odds of obtaining a positive SARS-CoV-2 test result using multivariate logistic regression models, is lower in IC versus immunocompetent individuals (18). In addition to severe COVID-19, IC populations are at higher risk of prolonged SARS-CoV-2 infection (19–26) and viral evolution (19–22, 24, 27, 28) due to poor humoral responses. These risks are exacerbated by even lower antibody responses to SARS-CoV-2 variants (29–35). High vaccine effectiveness is therefore critically important for this population, and the US Centers for Disease Control and Prevention (CDC) recommended a third dose of either mRNA-1273 or BNT162b2 COVID-19 vaccines as part of their primary series.

Influenza vaccine trials demonstrated that high-dose vaccines led to improved immune responses in IC individuals compared with standard dose vaccines and suggested that a high-dose vaccine offers greater effectiveness for IC populations (36–41). Although both mRNA-1273 and BNT162b2 employ the mRNA mode of action, the composition of each vaccine is different. For instance, the mRNA dosage and type of lipid nanoparticles used in the delivery system differs between vaccines. The mRNA-1273 primary series contains 100 mcg of mRNA and 50 mcg for the booster (2, 42), whereas BNT162b2 contains 30 mcg of mRNA for each primary and booster dose (3, 43). Observational studies have consistently shown differences between the 2 mRNA COVID-19 vaccines, both in terms of immune response (15) and clinical effectiveness (44–46) in IC populations.

As SARS-CoV-2 transitions from a pandemic to an endemic state, countries are transferring vaccination programs from central government purchasing to their respective national healthcare systems, which is triggering in-depth health technology assessments to recommend the best use of available vaccines in specific populations. Several national immunization technical advisory groups, including the Advisory Committee on Immunization Practices in the United States, use the GRADE (Grading of Recommendations, Assessment, Development and Evaluations) framework for identifying questions relevant to healthcare, selecting outcomes of interest and assessing their importance, evaluating the available evidence, and synthesizing evidence to develop recommendations consistent with considerations of values and preferences of patients and the society in which they live (47, 48).

This present analysis follows the GRADE framework to address the following healthcare question: Is the mRNA-1273 COVID-19 vaccine (50 or 100 mcg/dose) more clinically effective in IC populations compared with the BNT162b2 COVID-19 vaccine (30 mcg/dose)? Accordingly, we performed a systematic literature review and pairwise meta-analysis to compare COVID-19 vaccine effectiveness outcomes among IC individuals given either mRNA-1273 or BNT162b2.

## 2 Methods

### 2.1 Search strategy and study selection

We performed a systematic literature review in accordance with the Preferred Reporting Items for Systematic Reviews and Meta-Analyses 2020 framework (49). The main search was conducted in the World Health Organization COVID-19 Research Database on April 14, 2022, and updated on December 19, 2022. Databases searched were MEDLINE, International Clinical Trials Registry Platform, Embase, EuropePMC, medRxiv, Web of Science, ProQuest Central, Academic Search Complete, Scopus, and COVIDWHO. The search strategy is presented in **Table S1**.

Clinical trials, observational studies, or any real-world evidence published as manuscripts, letters, commentaries, abstracts, or posters were included if they reported efficacy or clinical effectiveness outcomes in IC individuals ≥18 years of age vaccinated with mRNA-1273 or BNT162b2 within the same study. IC individuals were defined as people with immunocompromising conditions considered to be in the clinically extremely vulnerable (CEV) groups 1 or 2, which includes solid organ transplant, solid and hematologic cancers, hemodialysis, poorly controlled HIV infection, and autoimmune conditions requiring immunosuppressive therapy (50). Outcomes of interest were vaccine efficacy or effectiveness against symptomatic, laboratory-confirmed SARS-CoV-2 infection, SARS-CoV-2 infection, severe SARS-CoV-2 infection (defined as patients with infection and hospitalization or death, or as reported by each study), COVID-19–associated hospitalization, and COVID-19–associated death. Recently published systematic literature reviews on the same topic were cross-checked to ensure relevant articles were included. Studies reporting outcomes in pregnant women, current or former smokers, physically inactive people, or those with a heterologous vaccination schedule (ie, mix of mRNA-1273 and BNT162b2), only safety data, or study protocols or economic models were excluded. The population, exposure, comparison, and outcomes used in the systematic literature review are summarized in **Table S2**. Two independent reviewers selected studies following a 2-level approach; a third reviewer arbitrated conflicts. Titles and abstracts were screened against inclusion criteria in level 1, followed by full-text appraisal of articles not excluded at level 1 against selection criteria in level 2.

### 2.2 Data extraction and quality assessment

Publication details, study and participant characteristics, vaccine type and vaccination status, at-risk condition, and clinical outcomes were extracted. Risk of bias (RoB) was assessed in accordance with Cochrane review guidelines (51) using the Newcastle-Ottawa Scale (52) for observational studies. Evidence was evaluated based on GRADE criteria (47, 48).

### 2.3 Statistical analysis

Random-effects meta-analysis models were used to pool risk ratios (RR) and calculate absolute effects as risk difference (RD) per 100,000 individuals across studies. Inverse variance weights were calculated for individual studies with the DerSimonian-Laird method (53). Chi-square testing to evaluate heterogeneity across studies was performed (54). The *I^2^* statistic was estimated (0%–100%, 0% meaning no evidence of heterogeneity). Subgroup analysis was performed for patients with cancer, autoimmune disease, and solid organ transplant to account for differences between the underlying health conditions in the overall IC population.

## 3 Results

### 3.1 Overview of included studies

**Figure 1:**
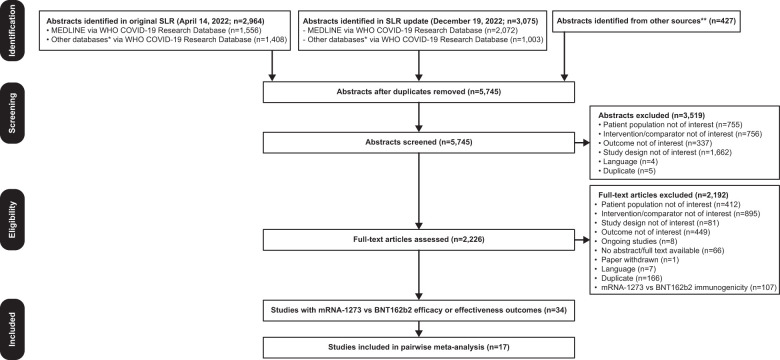
PRISMA Flow Diagram. Searches were first performed on April 14, 2022 followed by an update on December 19, 2022. *Databases searched include ICTRP, Embase, EuropePMC, medRxiv, Web of Science, ProQuest Central, Academic Search Complete, Scopus, and COVIDWHO. **Includes internal documents from Moderna and recently published SLRs. COVID-19, coronavirus disease 2019; PRISMA, Preferred Reporting Items for Systematic Reviews and Meta-Analyses; SLR, systematic literature review; WHO, World Health Organization.

Of 5745 unique items retrieved, 34 studies were identified reporting COVID-19 clinical efficacy or effectiveness outcomes in IC individuals ≥18 years of age who received mRNA-1273 or BNT162b2 in the same study (**Figure 1**). Seventeen articles were excluded because the population did not meet the inclusion criteria (ie, participants had IC conditions not included in CEV groups 1 or 2), 1-dose vaccine regimen data were reported, or the outcome of interest data were not reported in sufficient detail for analysis. Characteristics of all studies (n=17) included in the pairwise meta-analysis are shown in **Table 1**. Overall, 178,298 and 170,760 patients received mRNA-1273 and BNT162b2, respectively. Studies included mostly US populations (n=12) (18, 44, 46, 55–59, 61, 64, 65, 67), with the remaining trials reporting data on patients from Spain (n=2) (45, 62), Italy (n=1) (63), Singapore (n=1) (66), and multiple countries (n=1) (60). Specific at-risk and IC conditions included solid organ transplant (n=5) (44, 45, 55, 61, 67), cancer (n=5) (18, 44, 58, 63, 64), hemodialysis (n=2) (56, 65), rheumatologic disease (n=3) (18, 46, 60), multiple sclerosis or other neurologic autoimmune disease (n=2) (57, 66), inflammatory bowel disease (n=1) (59), and hematologic disorders (n=1) (62). Individuals received ≥2 doses of mRNA-1273 or BNT162b2. Data on 2-dose regimens were considered if reported (n=13) (18, 44, 45, 55–57, 59–62, 64, 65, 67); otherwise, data from 3- or 4-dose regimens (n=4) (46, 58, 63, 66) were used. Outcomes were assessed ≥14 days after the second dose (n=13) (18, 44, 45, 55, 56, 58, 60–64, 66, 67), other timepoints (n=2) (46, 65), and ≥7 days after the second dose (n=1) (59). Timing of outcome assessment relative to the second dose was not specified in 1 study (57). Variants of concern were delta (n=6) (18, 44, 45, 62, 64, 67), delta and omicron (n=2) (58, 66), delta and beta (n=1) (56), and pre-omicron variants (n=1) (46). Seven studies did not directly specify the variant assessed (55, 57, 59–61, 63, 65).

**Table 1:** Characteristics of studies included in the meta-analysis.

	Study Characteristics						Outcomes Reported				
Author, Year	Design	Data Source	Population	Vaccine	Study Period	Vaccinated, n	Symptomatic Infection	Infection	Severe Infection	Hospitalization	Death
Aslam, 2021 (55)	• Retrospective single-center cohort	Transplant registry	• USA • Solid organ transplant	• 2 doses (MM vs PP) • mRNA-1273 (100 mcg) • BNT162b2 (30 mcg)	Jan 1, 2021 – Feb 6, 2021	mRNA-1273: 632 BNT162b2: 375	Y	Y	Y	Y	Y
Butt, 2022 (56)	• Test-negative design • 1:1 Propensity-score matched analysis of cases and controls	Veterans Affairs	• USA • Chronic hemodialysis	• 2 doses (MM vs PP) • mRNA-1273 (100 mcg) • BNT162b2 (30 mcg)	Jan 2021 – Aug 2021	mRNA-1273: 630 BNT162b2: 719	N	Y	N	N	N
Embi, 2021 (18)	• Test-negative design	VISION Network	• USA • IC and immunocompetent vaccine recipients	• 2 doses (MM vs PP) • mRNA-1273 (100 mcg • BNT162b2 (30 mcg)	Jan 17, 2021 – Sep 5, 2021	**IC and vaccinated** mRNA-1273: 4337 BNT162b2: 6227 **Solid malignancy** mRNA-1273: 2053 BNT162b2: 2848 **Rheumatologic or inflammatory disorder** mRNA-1273: 1053 BNT162b2: 1591	N	Y	Y	Y	N
Holroyd, 2022 (57)	• Retrospective single-center study	CLIMB	• USA • Patients with MS on disease- modifying therapies vaccinated vs healthy controls	• 2 doses (MM vs PP) • mRNA-1273 (100 mcg) • BNT162b2 (30 mcg)	Jun 2021 – Dec 2021	mRNA-1273: 110 BNT162b2: 133	N	Y	Y	Y	Y
Kelly, 2022 (58)	• Retrospective cohort study	US VHA	• USA • IC patients including cancer	• 3 doses (MMM vs PPP) • mRNA-1273 (100 mcg and booster 50 mcg) • BNT162b2(30 mcg)	Jul 2021 – May 2022	mRNA-1273: 79,517 BNT162b2: 67,780	Y	Y	Y	Y	N
Khan, 2021 (59)	• Retrospective cohort study	US VHA	• USA • Patients with inflammatory bowel disease exposed to various conventional and advanced immuno-suppressive therapies	• 1 or 2 doses (MM vs PP) • mRNA-1273 (100 mcg) • BNT162b2 (30 mcg)	Dec 18, 2020 (index date) – Apr 20, 2021	**Fully vaccinated** mRNA-1273: 3380 BNT162b2: 2873	N	Y	Y	N	N
Liew, 2022 (60)	• Retrospective registry study	REDcap	• Multicountry, with 65% of the patient population from North America • Rheumatic disease	• 2 doses (MM vs PP) • mRNA-1273 (100 mcg) • BNT162b2 (30 mcg)	Jan 2021 – Sep 2021	mRNA-1273: 45 BNT162b2: 21	N	N	Y	Y	Y
Malinis, 2021 (61)	• Retrospective observational study	Yale New Haven chart review	• USA • Solid organ transplant recipients	• 2 doses (MM vs PP) • mRNA-1273 (100 mcg) • BNT162b2 (30 mcg)	As of May 18, 2011 (start date not reported)	mRNA-1273: 157 BNT162b2: 275	N	Y	Y	N	Y
Mazuecos, 2022 (45)	• Retrospective national cohort study	National registry of patients with kidney transplantation	• Spain • Kidney transplant recipients	• 2 doses (MM vs PP) • mRNA-1273 (100 mcg) • BNT162b2(30 mcg)	Apr 2021 – Oct 2021	mRNA-1273: 213 BNT162b2: 121	N	N	Y	Y	Y
Mues, 2022 (44)	• Observational comparative effectiveness study • 1:1 Propensity-score matched on age, sex, payer type, state of residence, previous healthcare use, comorbidities, frailty score and IC group	US insurance claims	• USA • IC individuals	• 2 doses(MM vsPP) • mRNA-1273 (100mcg) • BNT162b2 (30 mcg)	Dec 11, 2020 – Jan 10, 2022	**IC** mRNA-1273: 57,000 BNT162b2: 66,757 **Solid organ transplant** mRNA-1273: 4029 BNT162b2: 5043 **Active cancer** mRNA-1273: 7186 BNT162b2: 8277	N	Y	Y	Y	N
Patel, 2022 (46)	• Retrospective cohort study	MGB healthcare system	• USA • Rheumatic disease	• ≥2 doses (MMM vs PPP) • mRNA-1273 (100 mcg and booster 50 mcg) • BNT162b2 (30 mcg)	Nov 2021 – Dec 2022	mRNA-1273: 4588 BNT162b2: 6080	N	Y	N	N	N
Piñana, 2022 (62)	• Prospective multicenter registry-based cohort study	GRUCINI with SEHH	• Spain • Patients with hematologic disorders	• 2 doses (MM vs PP) • mRNA-1273 (100 mcg) • BNT162b2 (30 mcg)	Dec 2020 – Dec 2021	mRNA-1273: 982 BNT162b2: 362	N	Y	N	N	N
Pino, 2022 (63)	• Retrospective cohort study	Patients followed up at Medical Oncology Unit in Florence at Santa Maria Annunziata, Serristori and Borgo San Lorenzo Hospitals	• Italy • Extremely vulnerable individuals, patients with cancer on systemic antitumor treatment	• 3 doses (MMM vs PPP) • mRNA-1273 (100 mcg and booster 50 mcg) • BNT162b2 (30 mcg)	Mar 26, 2021 – Apr 4, 2021	mRNA-1273: 527 BNT162b2: 96	N	Y	N	N	N
Rooney, 2022 (64)	• Retrospective study	The University of Kansas Cancer Center Curated Cancer Clinical Outcomes Database	• USA • Patients with cancer receiving antineoplastic therapy	• 2 doses (MM vs PP) • mRNA-1273 (100 mcg) • BNT162b2 (30 mcg)	Feb 2021 – Oct 2021	mRNA-1273: 2993 BNT162b2: 6423	N	Y	N	N	N
Sibbel, 2021 (65)	• Retrospective observational study	Dialysis center	• USA • Hemodialysis patients	• 1 or 2 doses (MM vs PP) • mRNA-1273 (100 mcg) • BNT162b2 (30 mcg)	Study duration: 4 months	mRNA-1273: 23,037 BNT162b2: 12,169	N	Y	Y	Y	Y
Yeo, 2022 (66)	• Prospective, observational study	NR	• Singapore • Patients with MS, AQP4-NMOSD, and MOGAD	• 2−3 doses (MMM vs PPP) • mRNA-1273 (100 mcg and booster 50 mcg) • BNT162b2 (30 mcg)	Study cut-off date: Dec 31, 2022	mRNA-1273: 38 BNT162b2: 327	Y	Y	Y	Y	N
Yetmar, 2022 (67)	• Retrospective cohort	From 1 US center	• USA • Solid organ transplant recipients	• 2 doses (MM vs PP) • mRNA-1273 (100 mcg) • BNT162b2 (30 mcg)	Aug 2021 – Sep 2021	mRNA-1273: 12 BNT162b2: 22	Y	Y	Y	N	Y

AQP4-NMOSD, aquaporin-4-antibody neuromyelitis optica spectrum disorder; CLIMB, Comprehensive Longitudinal Investigation of Multiple Sclerosis at Brigham and Women’s Hospital; GRUCINI, Infectious Complications Subcommittee of the Spanish Hematopoietic Stem Cell Transplantation and Cell Therapy Group; IC, immunocompromised; MGB, Mass General Brigham; MOGAD, myelin oligodendrocyte glycoprotein-antibody−associated disease; MS, multiple sclerosis; NR, not reported; SEHH, Spanish Society of Hematology and Hemotherapy; VHA, Veterans Health Administration.

Vaccine dosing was abbreviated as MM or MMM for 2 or 3 doses of mRNA-1273, respectively, and as PP or PPP for 2 or 3 doses of BNT162b2, respectively.

Risk-of-bias assessment found no serious RoB for 13 studies and serious RoB in 4 nonrandomized studies, primarily because of the lack of description of comparability between cohorts or adjustment for confounding factors (**Table S3**).

### 3.2 Symptomatic, laboratory-confirmed SARS-CoV-2 infection

Of the 4 studies reporting symptomatic, laboratory-confirmed SARS-CoV-2 infection, mRNA COVID-19 vaccine type was not associated with a statistically significant risk of infection (RR, 0.88 [95% CI, 0.65–1.19]; **Table 2**). The RD (95% CI) of mRNA-1273 compared with BNT162b2 was estimated to be 633 fewer infections (792 fewer to 473 fewer). Heterogeneity between studies analyzed for this outcome may be considered substantial (*I^2 ^= *60.3%). Evidence certainty was rated as type 3 (low) due to the inclusion of nonrandomized studies and because of imprecision (**Table 2**).

**Table 2:** GRADE summary of findings overall.

Certainty Assessment							mRNA-1273, n/N, (%)	BNT162b2, n/N, (%)	Effect Relative (95% CI)	Effect Absolute (95% CI)	Certainty
Studies, n	Study Design	RoB	Inconsistency	Indirectness	Imprecision	Other Considerations					
Symptomatic, laboratory confirmed SARS-CoV-2 infection											
4	NR	Not serious	Serious^a^	Not serious	Very serious^b^	None	1790/ 80,199 (2.23)	1990/ 68,504 (2.90)	**RR 0.88** (0.65− 1.19)	633 fewer per 100,000*** (from 792 fewer to 473 fewer)	Type 3^c^
SARS-CoV-2 infection											
15	NR	Not serious	Serious^d^	Serious^e^	Serious^f^	Strong association	3038/ 155,399 (1.95)	3559/ 145,697 (2.44)	RR 0.85* (0.75− 0.97)	375 fewer per 100,000** (from 626 fewer to 124 fewer)	Type 4^g^
Severe SARS-CoV-2 infection											
12	NR	Not serious	Not serious^h^	Serious^i^	Serious^j^	Strong association	564.5/ 145,372 (0.39)	649.5/ 131,379 (0.49)	RR 0.85*** (0.77–0.93)	**25 fewer per 100,000** (from 82 fewer to 32 more)	Type 4^k^
Hospitalization due to COVID-19											
9	NR	Not serious	Not serious^l^	Not serious^m^	Serious^n^	Strong association	514.5/ 141,821 (0.36)	570.5/ 128,207 (0.44)	RR 0.88*** (0.79− 0.97)	**14 fewer per 100,000** (from 78 fewer to 51 more)	Type 3^c^
Death due to COVID-19											
7	NR	Not serious	Not serious°	Not serious^m^	Serious^p^	Strong association	53/24,186 (0.22)	49/13,144 (0.37)	RR 0.63* (0.44− 0.90)	**15 fewer per 100,000** (from 307 fewer to 227 more)	Type 3^c^

COVID-19, coronavirus disease 2019; GRADE, Grading of Recommendations, Assessment, Development and Evaluations; NR, nonrandomized studies; RoB, risk of bias; RR, risk ratio; SARS-CoV-2, severe acute respiratory syndrome coronavirus 2.

*P<0.05; **P<0.01; ***P<0.001.

a *I^2^* =60.3%, *X^2^* =7.56, p(Q)=0.06, substantial heterogeneity.

b In Aslam 2021, only 2 events each occurred in the mRNA-1273 and the BNT162b2 arms, therefore wide 95% CI.

c Lower grading due to imprecision. Type 3 due to inclusion of nonrandomized studies.

d *I^2^* =67.7%, *X^2^* =43.40, p(Q)<0.0001, substantial heterogeneity.

e Outcome definitions rather heterogeneous (test-positive cases and symptomatic cases).

f In Holroyd 2022, Malinis 2021, and Yeo 2022, only 1 event occurred in the mRNA-1273 arm; in Pino 2022, only 1 event occurred in the BNT162b2 arm. The small number of events resulted in a wider 95% CI.

g Lower grading due to imprecision and indirectness because of varying outcome definitions (symptomatic and not further described SARS-CoV-2 infection)

h *I^2^* =0%, *X^2^* =6.46, p(Q)=0.84, no issues of heterogeneity and inconsistency.

i Outcome definitions rather heterogeneous (defined as severe infection, hospitalization, or death following SARS-CoV-2 infection).

j In Holroyd 2022, Malinis 2021, and Yeo 2022, only 0 or 1 event in the mRNA-1273 arm, which required a continuity correction of 0.5 for 0 events. The continuity correction resulted in a wider 95% CI.

k Lower grading because of imprecision and indirectness due to varying outcome definitions (severe SARS-CoV-2 infection and hospitalization or death following SARS-CoV-2 infection).

l *I^2^* =0%, *X^2^* =5.05, p(Q)=0.75, no issues of heterogeneity and inconsistency.

m No indirect comparisons, outcome definitions in line.

n In Holroyd 2022 and Yeo 2022, only 0 and 1 event in the mRNA-1273 arm, respectively; this resulted in a wider 95% CI.

°*I^2^* =0%, *X^2^* =2.12, p(Q)=0.91, no issues of heterogeneity and inconsistency.

p In Yetmar 2022, the sample size was rather low and 0 events occurred in both arms; therefore, a continuity correction of 0.5 was necessary. A continuity correction was also necessary in Aslam 2021 and Holroyd 2022 because 0 events occurred in both arms. This resulted in a wide 95% CI.

Subgroup analysis was performed in 2 studies of solid organ transplant recipients (55, 67) and in 1 study of patients with autoimmune disease (66). No association between mRNA COVID-19 vaccine type and risk of symptomatic, laboratory-confirmed SARS-CoV-2 infection was observed in either the solid organ transplant recipient subgroup (RR, 1.11 [95% CI, 0.86–1.43]; RD, −202 [95% CI, −1059 to 655]) or the autoimmune disease subgroup (RR, 0.48 [95% CI, 0.07–3.48; RD, −2873 [95% CI, −8531 to 2785]; **Table 3**).

**Table 3:** GRADE summary of findings by population subgroup.

Certainty Assessment							mRNA-1273, n/N, (%)	BNT 162b2, n/N, (%)	Effect Relative (95% CI)	Effect Absolute (95% CI)	Certainty
Studies, n	Study Design	RoB	Inconsistency	Indirectness	Imprecision	Other Considerations					
Symptomatic, laboratory-confirmed SARS-CoV-2 infection											
Solid organ transplant											
2	NR	Not serious	Not serious^a^	Not serious	Serious^b^	None	13/644 (2.02)	20/397 (5.04)	**RR 1.11** (0.86–1.43)	**202 fewer per 100,000** (from 1059 fewer to 655 more)	Type 4^c^
Autoimmune disease											
1	NR	Not serious	Not serious^d^	Not serious	Very serious^e^	None	1/38 (2.63)	18/327 (5.50)	**RR 0.48** (0.07–3.48)	**2873 fewer per 100,000** (from 8531 fewer to 2785 more)	Type 4^c^
SARS-CoV-2 infection											
Solid organ transplant											
4	NR	Not serious	Not serious^f^	Serious^g^	Very serious^h^	None	106/ 4830 (2.19)	139/ 5715 (2.43)	**RR 1.05** (0.87–1.26)	**93 fewer per 100,000** (from 573 fewer to 386 more)	Type 4^i^
Cancer											
5	NR	Not serious	Not serious^j^	Serious^g^	Very serious^k^	Strong association	146/ 13,741 (1.06)	266/ 18,006 (1.48)	RR 0.68*** (0.55− 0.83)	437 fewer per 100,000** (from 699 fewer to 175 fewer)	Type 4^i^
Autoimmune disease											
5	NR	Not serious	Not serious^l^	Serious^g^	Very serious^m^	Strong association	127/ 9169 (1.39)	242/ 11,004 (2.20)	RR 0.77* (0.62− 0.96)	**363 fewer per 100,000** (from 956 fewer to 231 more)	Type 4^i^
Severe SARS-CoV-2 infection											
Solid organ transplant											
5	NR	Not serious	Not serious^n^	Serious°	Very serious^p^	None	164/ 5045 (3.25)	124/ 5838 (2.12)	**RR 0.91** (0.79–1.06)	**16 fewer per 100,000** (from 275 fewer to 244 more)	Type 4^q^
Cancer											
2	NR	Not serious	Not serious^r^	Serious°	Not serious	Strong association	38/ 9239 (0.41)	94/ 11,125 (0.84)	RR 0.54** (0.37–0.79)	**585 fewer per 100,000** (from 1655 fewer to 485 more)	Type 4^s^
Autoimmune disease											
5	NR	Not serious	Not serious^t^	Serious°	Very serious^u^	None	56.5/ 4603 (1.23)	94.5/ 4970 (1.90)	**RR 0.98** (0.71–1.36)	**24 more per 100,000** (from 82 fewer to 130 more)	Type 4^q^
Hospitalization due to COVID-19											
Solid organ transplant											
3	NR	Not serious	Not serious^v^	Not serious^w^	Serious^x^	None	163/ 4874 (3.34)	120/ 5539 (2.17)	**RR 0.91** (0.79− 1.06)	**31 fewer per 100,000** (from 330 fewer to 268 more)	Type 4^y^
Cancer											
2	NR	Not serious	Not serious^z^	Not serious^w^	Not serious	Strong association	38/ 9239 (0.41)	94/ 11,125 (0.84)	RR 0.54** (0.37− 0.79)	**585 fewer per 100,000** (from 1655 fewer to 485 more)	Type 3^aa^
Autoimmune disease											
4	NR	Not serious	Not serious^bb^	Not serious^w^	Serious^cc^	None	54.5/ 1223 (4.46)	93.5/ 2097 (4.46)	**RR 0.98** (0.70− 1.35)	**16 fewer per 100,000** (from 1139 fewer to 1107 more)	Type 3^dd^
Death due to COVID-19											
Solid organ transplant											
4	NR	Not serious	Not serious^ee^	Not serious^w^	Very serious^ff^	Strong association	36.5/1017 (3.59)	37.5/796 (4.71)	RR 0.57** (0.38− 0.85)	**180 fewer per 100,000** (from 1443 fewer to 1083 more)	Type 3^gg^
Autoimmune disease											
2	NR	Not serious	Not serious^hh^	Not serious^w^	Very serious^ii^	None	1.5/ 132 (1.14)	3.5/ 179 (1.96)	**RR 0.81** (0.12–5.53)	**40 more per 100,000** (from 1563 fewer to 1642 more)	Type 4^jj^

COVID-19, coronavirus disease 2019; GRADE, Grading of Recommendations, Assessment, Development and Evaluations; NR, nonrandomized studies; RoB, risk of bias; RR, risk ratio; SARS-CoV-2, severe acute respiratory syndrome coronavirus 2.

*P<0.05; **P<0.01; ***P<0.001.

a *I^2^* =0%, *X^2^* =0.4, p(Q)=0.53, no issues of heterogeneity and inconsistency.

b In Aslam 2021, only 2 events occurred in the mRNA-1273 and BNT162b2 arms. The small number of events resulted in a wide 95% CI.

c Lower grading due to imprecision and limited evidence.

d *I^2^* =0%, *X^2^* =0, p(Q)=1, no issues of heterogeneity and inconsistency.

e In Yeo 2022, only 1 event occurred in the mRNA-1273 arm. The small number of events resulted in a wide 95% CI.

f *I^2^* =0%, *X^2^* =0.81, p(Q)=0.85, no issues of heterogeneity and inconsistency.

g Outcome definitions were rather heterogeneous (test-positive cases and symptomatic cases).

h In Aslam 2021 and Malinis 2021, 2 events and 1 event occurred in the mRNA-1273 arm, respectively. There were additionally 2 events in the BNT162b2 arms. The small number of events resulted in a wide 95% CI.

i Lower grading because of imprecision and indirectness due to varying outcome definitions (symptomatic and not further described SARS-CoV-2 infection).

j *I^2^* =0%, *X^2^* =1.45, p(Q)=0.84, no issues of heterogeneity and inconsistency.

k In Pino 2022, only 1 event in the BNT162b2 arm; this resulted in a wider 95% CI.

l *I^2^* =0%, *X^2^* =3.21, p(Q)=0.52, no issues of heterogeneity and inconsistency.

m In Holroyd 2022 and Yeo 2022, only 1 event occurred in the mRNA-1273 arm. The small number of events resulted in a wide 95% CI.

n *I^2^* =0%, *X^2^* =0.72, p(Q)=0.95, no issues of heterogeneity and inconsistency.

°Outcome definitions were rather heterogeneous (severe infection defined directly, or COVID-19–related hospitalization or death).

p In Aslam 2021 and Malinis 2021, a continuity correction of 0.5 was applied because there were 0 events in the mRNA-1273 and BNT162b2 arms. This resulted in a wide 95% CI.

q Lower grading because of imprecision and indirectness due to varying outcome definitions (severe infection defined directly, or COVID-19–related hospitalization or death).

r *I^2^* =0%, *X^2^* =0.02, p(Q)=0.89, no issues of heterogeneity and inconsistency.

s Lower grading due to limited evidence and indirectness due to varying outcome definitions (severe infection defined directly, or COVID-19–related hospitalization or death).

t *I^2^* =0%, *X^2^* =0.26, p(Q)=0.99, no issues of heterogeneity and inconsistency.

u In Holroyd 2022, a continuity correction of 0.5 was applied because there were 0 events in the mRNA-1273 and BNT162b2 arms. There was additionally only 1 event in the mRNA-1273 arm of Yeo 2022. This resulted in a wide 95% CI.

v *I^2^* =0%, *X^2^* =0.24, p(Q)=0.89, no issues of heterogeneity and inconsistency.

w No indirect comparisons, outcome definitions in line.

x In Aslam 2021, 1 event occurred in the mRNA-1273 and BNT162b2 arms. The small number of events resulted in a wide 95% CI.

y Lower grading due to imprecision and limited evidence.

z *I^2^* =0%, *X^2^* =0.02, p(Q)=0.89, no issues of heterogeneity and inconsistency.

^aa^Lower grading due to limited evidence, higher grading due to no issues with imprecision and strong association.

^bb^ *I^2^* =0%, *X^2^* =0.06, p(Q)=1, no issues of heterogeneity and inconsistency.

^cc^In Holroyd 2022, a continuity correction of 0.5 was applied because there were 0 events in the mRNA-1273 and BNT162b2 arms. This resulted in a wide 95% CI.

^dd^Higher grading due to no issues with indirectness, lower grading due to no strong association.

^ee^ *I^2^* =0%, *X^2^* =0.67, p(Q)=0.88, no issues of heterogeneity and inconsistency.

^ff^In Aslam 2021, Malinis 2021, and Yetmar 2022, a continuity correction of 0.5 was applied because there were 0 events in the mRNA-1273 and BNT162b2 arms. This resulted in a wide 95% CI.

^gg^Lower grading due to imprecision, higher grading due to no issues with indirectness and strong association.

^hh^ *I^2^* =0%, *X^2^* =0.05, p(Q)=0.82, no issues of heterogeneity and inconsistency.

^ii^In Holroyd 2022, a continuity correction of 0.5 was applied because there were 0 events in the mRNA-1273 and BNT162b2 arms. This resulted in a wide 95% CI.

^jj^Lower grading due to limited evidence, imprecision, and no strong association.

### 3.3 SARS-CoV-2 infection

Meta-analysis of the 15 studies reporting SARS-CoV-2 infection in IC populations showed that mRNA-1273 was associated with a statistically significant reduction in the risk of SARS-CoV-2 infection compared with BNT162b2 (RR, 0.85 [95% CI, 0.75–0.97]; *P*=0.0151). The RD (95% CI) of mRNA-1273 versus BNT162b2 was estimated to be 375 fewer SARS-CoV-2 infections (from 626 fewer to 124 fewer; *P*=0.0034). Heterogeneity between studies may be considered substantial (*I^2 ^= *67.7%). The certainty of evidence was graded as type 4 (very low) for imprecision and indirectness due to varying outcome definitions (**Table 2**).

Analysis of 5 studies reporting SARS-CoV-2 infection in patients with cancer (18, 44, 62–64) found that mRNA-1273 was associated with significantly reduced risk of infection compared with BNT162b2 (RR, 0.68 [95% CI, 0.55–0.83]; *P*=0.0002; RD, −437 [95% CI, −699 to −175]; *P*=0.0011). Similar findings were observed in 5 studies assessing patients with autoimmune diseases (RR, 0.77 [95% CI, 0.62–0.96]; *P*=0.0187; RD, −363 [95% CI, −956 to 231]) (18, 46, 57, 59, 66). No association between mRNA vaccine type and risk of infection was found for the 4 studies reporting SARS-CoV-2 infection in solid organ transplant recipients (RR, 1.05 [95% CI, 0.87–1.26]; RD, −93 [95% CI, −573 to 386]) (44, 55, 61, 67). No evidence of heterogeneity was observed between any of the studies (*I^2 ^= *0% for all subgroups). As in the overall meta-analysis of SARS-CoV-2 infection, the certainty of evidence was graded as type 4 (very low; **Table 3**).

### 3.4 Severe SARS-CoV-2 infection

Compared with BNT162b2, mRNA-1273 was associated with a statistically significant reduction in the risk of severe SARS-CoV-2 infection among IC patients in 12 studies (RR, 0.85 [95% CI, 0.77–0.93]; *P*=0.0009). The RD (95% CI) of mRNA-1273 versus BNT162b2 was 25 fewer severe SARS-CoV-2 infections (from 82 fewer to 32 more). No evidence of heterogeneity was observed between studies (*I^2 ^= *0%). The certainty of evidence for this outcome was type 4 (very low) because of imprecision and indirectness due to varying outcome definitions (**Table 2**).

In 2 studies reporting severe SARS-CoV-2 infection in patients with cancer (18, 44), mRNA-1273 was associated with a statistically significant reduction in the risk of severe infection compared with BNT162b2 (RR, 0.54 [95% CI, 0.37–0.79]; *P*=0.0013; RD, −585 [95% CI, −1655 to 485]). No evidence of heterogeneity was observed between studies (*I^2 ^= *0%). The certainty of evidence for severe SARS-CoV-2 infection in this subgroup was type 4 (very low) due to limited evidence and indirectness due to varying outcome definitions. mRNA COVID-19 vaccine type was not associated with statistically significant differences in the risk of severe SARS-CoV-2 infection in solid organ transplant (44, 45, 55, 61, 67) (RR, 0.91 [95% CI, 0.79–1.06]; RD, −16 [95% CI, −275 to 244]) and autoimmune disease subgroups (18, 57, 59, 60, 66) (RR, 0.98 [95% CI, 0.71–1.36]; RD, 24 [95% CI, −82 to 130]; **Table 3**).

### 3.5 Hospitalization due to COVID-19

mRNA-1273 was associated with a significantly lower risk of COVID-19–associated hospitalization versus BNT162b2 in the 9 studies included in the overall analysis (RR, 0.88 [95% CI, 0.79–0.97]; *P*<0.0001). The RD (95% CI) of mRNA-1273 compared with BNT162b2 was estimated to be 14 fewer hospitalizations due to COVID-19 (from 78 fewer to 51 more). No evidence of heterogeneity was observed between studies (*I^2 ^= *0%). The certainty of evidence for this outcome was type 3 (low) because of inclusion of nonrandomized studies and imprecision (**Table 2**).

In 2 studies reporting hospitalization in patients with cancer (18, 44), mRNA-1273 was associated with a significantly reduced risk of hospitalization compared with BNT162b2 (RR, 0.54 [95% CI, 0.37–0.79]; *P*=0.0013; RD, −585 [95% CI, −1655 to 485]). No association between mRNA vaccine type and COVID-19–associated hospitalization was found for the 4 studies each reporting hospitalization in the subgroups of patients with autoimmune diseases (RR, 0.98 [95% CI, 0.70–1.35]; RD, −16 [95% CI, −1139 to 1107]) (18, 57, 60, 66) or solid organ transplant (RR, 0.91 [95% CI, 0.79–1.06]; RD, −31 [95% CI, −330 to 268]) (44, 45, 61). No evidence of heterogeneity was observed between any of the studies for the subgroup analysis (*I^2 ^= *0%). The certainty of evidence in the cancer subgroup was graded as type 3 (low; **Table 3**).

### 3.6 Death due to COVID-19

Of the 7 studies reporting COVID-19–associated mortality (45, 55, 57, 60, 61, 65, 67), mRNA-1273 was associated with a significantly reduced risk of death compared with BNT162b2 (RR, 0.63 [95% CI, 0.44–0.90]; *P*=0.0119). Vaccination with mRNA-1273 was estimated to result in 15 fewer deaths associated with COVID-19 (from 307 fewer to 227 more) compared with BNT162b2. No evidence of heterogeneity was observed between the studies (*I^2 ^= *0%). The certainty of evidence was rated as type 3 (low) due to inclusion of nonrandomized studies (**Table 2**). Grading was reduced for imprecision.

COVID-19–associated death was assessed only in the subgroup of solid organ transplant recipients (n=4) (45, 55, 61, 67) and autoimmune disease (n=2) (57, 60).mRNA-1273 was associated with a significantly reduced risk of death compared with BNT162b2 in solid organ transplant recipients (RR, 0. 57 [95% CI, 0.38–0.85]; *P*=0.0056; RD, −180 [95% CI, −1443 to 1083]). No evidence of heterogeneity was observed between any of the studies in this subgroup (*I^2 ^= *0%). The certainty of evidence in the solid organ transplant subgroup was type 3 (low) because of inclusion of nonrandomized studies. Lower grading due to imprecision was offset by no concerns with indirectness and a strong association. mRNA COVID-19 vaccine type was not associated with statistically significant reduction in COVID-19–associated mortality in patients with autoimmune disease (RR, 0.81 [95% CI, 0.12–5.53]; RD, 40 [95% CI, −1563 to 1642]; **Table 3**).

## 4 Discussion

**Figure 2:**
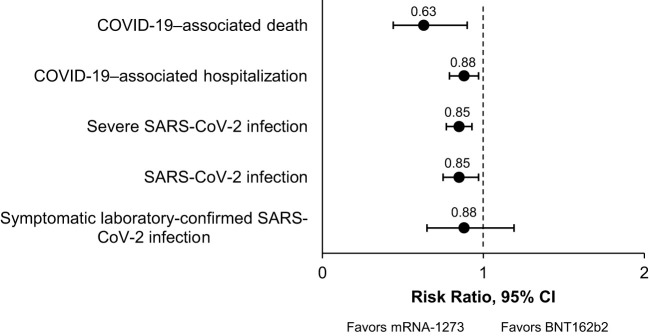
Summary of the Clinical Effectiveness Meta-Analysis. A graphical summary of the relative risks of clinical effectiveness outcomes analyzed for mRNA-1273 vs BNT162b2 COVID-19 vaccines in IC populations is shown. COVID-19, coronavirus disease 2019; IC, immunocompromised; SARS-CoV-2, severe acute respiratory syndrome coronavirus 2.

In this systematic review and pairwise meta-analysis of 17 studies, we found that mRNA-1273 was associated with a significantly lower risk of SARS-CoV-2 infection, severe SARS-CoV-2 infection, hospitalization due to COVID-19, and COVID-19–associated mortality compared with BNT162b2 in adults with a broad spectrum of severe IC conditions. A summary of the RR of each outcome for mRNA-1273 versus BNT162b2 is presented in **Figure 2**. The certainty of this evidence was type 4 (very low) for the SARS-CoV-2 infection and severe SARS-CoV-2 infection outcomes and type 3 (low) for the COVID-19–associated hospitalization and death outcomes (**Table 4**). Because all included studies were pairwise comparisons between mRNA-1273 and BNT162b2, the research question was not biased by differences in period assessed, population, or viral variants within each study. When outcomes were assessed in subgroups, mRNA-1273 was associated with significantly lower risk of SARS-CoV-2 infection, severe SARS-CoV-2 infection, and COVID-19–associated hospitalization versus BNT162b2 in patients with cancer. Compared with BNT162b2, mRNA-1273 was also associated with a significantly reduced risk of SARS-CoV-2 infection in patients with autoimmune diseases and COVID-19–associated death in solid organ transplant recipients.

**Table 4:** Summary of evidence for outcomes of interest.

Outcome	Outcome Importance^a^	Included in Evidence Profile	Certainty
Symptomatic, laboratory-confirmed SARS-CoV-2 infection	Critical	Yes	Type 3 (low)
SARS-CoV-2 infection	Critical	Yes	Type 4 (very low)
Severe SARS-CoV-2 infection	Critical	Yes	Type 4 (very low)
Hospitalization due to COVID-19	Critical	Yes	Type 3 (low)
Death due to COVID-19	Critical	Yes	Type 3 (low)

COVID-19, coronavirus disease 2019; GRADE, Grading of Recommendations, Assessment, Development, and Evaluations; SARS-CoV-2, severe acute respiratory syndrome coronavirus 2.

a Relative importance of outcomes assessed and ranked per the GRADE framework.

Immunocompromised individuals have a high burden of COVID-19 due to characteristics of their underlying disease or immunosuppressive treatments that affect their ability to mount productive immune responses and increased susceptibility to severe COVID-19 (30). Physicians may seek to optimize COVID-19 vaccine type, timing, and number of doses to improve outcomes in IC patients (32). RCTs are ranked highly in the hierarchy of evidence; however, studying comparative efficacy with adequate power would require enrolling a prohibitive number of IC patients. Given the rapid spread of SARS-CoV-2 globally, there was also insufficient time following the development of mRNA COVID-19 vaccines to conduct a larger number of RCTs in IC populations. Therefore, the research question can only be assessed using large real-world databases where individual medical and pharmacy information is available and real-world studies evaluating vaccine effectiveness in IC patients. Given the risk of severe COVID-19 in IC patients, our approach may be justified given the absence of RCT data in this vulnerable population.

Limitations of this systematic literature review were that non-English studies were excluded, and publication bias was not assessed in the meta-analysis. Inherent to the GRADE framework, evidence certainty is initially set to either high if the included studies are randomized studies or low if they are observational studies. Because all 17 studies included in the pairwise meta-analysis were nonrandomized, the maximum certainty of evidence achievable in this meta-analysis was low despite the high number of observational studies and consistency of results. The pairwise meta-analysis was also limited by inconsistent outcome definitions across studies and differences in covariates between studies. For example, the vaccination scheme (2 vs 3 doses; booster) differed between studies, with a mix of primary series (100 mcg vs 30 mcg) and booster (50 mcg vs 30 mcg) pairwise comparisons included in the meta-analysis. Variants of concern changed over time, with risks of hospitalization and death (68) and vaccine effectiveness differing by variant (69). Vaccine effectiveness of 2-dose regimens could only be shown for the ancestral strain through the delta variants, whereas the omicron variant required a 3-dose schedule. Other sources of bias, such as prescribing differences by risk of severe COVID-19, ability of patients to choose the mRNA vaccine type, and differing risks of infection or COVID-19–associated morbidity and mortality among patients with different IC conditions, could not be accounted for in this meta-analysis. In addition to differences in mRNA dosage between mRNA-1273 and BNT162b2, other differences such as the lipid nanoparticle delivery system and mRNA translation efficiency may also have impacted clinical effectiveness between vaccines.

Our meta-analysis of observational studies showed that mRNA-1273 (50 or 100 mcg/dose) was associated with a significantly reduced risk of SARS-CoV-2 infection, severe SARS-CoV-2 infection, COVID-19–associated hospitalization, and death due to COVID-19 when compared with BNT162b2 (30 mcg/dose) in IC populations. Based on these findings, vaccinating IC individuals in the United States with mRNA-1273 instead of BNT162b2 would prevent an additional 14 and 15 hospitalizations and deaths per 100,000 individuals, respectively. Although the numerical differences in estimated prevented COVID-19–associated hospitalizations and deaths are small, the implications for care are considerable in IC patients, who are at increased risk of severe COVID-19. Considering the limited availability of data from RCTs and to provide needed clinical decision-making guidance, our results showed that mRNA-1273 offers better clinical outcomes compared with BNT162b2 in vulnerable IC populations. A video summary linked to this article can be found at **Video S1**.

## Data availability statement

The original contributions presented in the study are included in the article/**Supplementary Material**. Further inquiries can be directed to the corresponding author.

## Author contributions

XW and KH designed and performed the systematic literature review and meta-analysis and critically evaluated the manuscript. AS, LP, AR, and AK designed and performed the systematic literature review and critically evaluated the manuscript. PS and SV collected data and critically evaluated the manuscript. MB-J and NV conceptualized the article and provided oversight and critical evaluation of the manuscript. All authors contributed to the article and approved the submitted version.
