# Supplementary material for: Comparative effectiveness of mRNA-1273 and BNT162b2 COVID-19 vaccines in immunocompromised individuals: a systematic review and meta-analysis using the GRADE framework
Source: Front Immunol. 2023 Sep 12;14:1204831. doi: 10.3389/fimmu.2023.1204831 (PMC10523015; doi:10.3389/fimmu.2023.1204831)
Supplement: Supplementary file 2 [file Table_2.docx]

Supplementary Material

Comparative Effectiveness of mRNA-1273 and BNT162b2 COVID-19 Vaccines in Immunocompromised Individuals: A Systematic Review and Meta-Analysis Using the GRADE Framework

**Xuan Wang, MD, MSc, Katrin Haeussler, PhD, MSc, Anne Spellman, PhD, MSc, Leslie E. Phillips, PhD, SM, Allison Ramiller, MPH, Mary T. Bausch-Jurken, PhD, MS, Pawana Sharma, MSc, Anna Krivelyova, MA, Sonam Vats, MPH, Nicolas Van de Velde, PhD***

*** Correspondence:** Corresponding Author: [Nicolas.VandeVelde@modernatx.com](mailto:Nicolas.VandeVelde@modernatx.com)

## Table S2. Research Question and PECOS

| **Research question** | Is the mRNA-1273 COVID-19 vaccine (50 or 100 mcg/dose) more clinically effective in IC populations compared with the BNT162b2 COVID-19 vaccine (30 mcg/dose)? | |
| --- | --- | --- |
|  | **Include** | **Exclude** |
| **Population** | IC individuals ≥18 years of age defined as people with CEV groups 1 and 2 medical conditions (51) | - Pregnant women, current/former smokers, physically inactive - Studies on only healthy individuals or individuals not categorized as CEV |
| **Exposure** | mRNA-1273 | Studies with heterologous vaccination schedule (i.e., data on mixed mRNA-1273, BNT162b2, or other vaccines) |
| **Comparison** | BNT162b2 |  |
| **Outcomes** | - Vaccine efficacy/effectiveness against COVID-19 infection - Vaccine efficacy/effectiveness against symptomatic COVID-19 - Vaccine efficacy/effectiveness against severe COVID-19 - Vaccine efficacy/effectiveness against hospitalization - Vaccine efficacy/effectiveness against death - SARS-CoV2 positivity (symptomatic or asymptomatic) - Symptomatic laboratory-confirmed COVID-19 infection - Severe COVID-19 infection (hospitalization or death) - Breakthrough infection - Hospitalization due to COVID-19 (ICU, ER, ventilation, etc) - Death due to COVID-19 | Studies only with safety results |
| **Study design** | - Clinical trials - Observational studies - Any kind of real-world evidence | - Study protocol (no results) - Economic models |
| **Other limits** | - Any publication type (including letters, commentary, abstract, full text, poster) - Publication in English |  |

CEV, clinically extremely vulnerable; COVID-19, coronavirus disease 2019; ER, emergency room; IC, immunocompromised; ICU, intensive care unit; PECOS, population, exposure, comparison, outcomes, and study design.
