# Supplementary material for: Comparative effectiveness of mRNA-1273 and BNT162b2 COVID-19 vaccines in immunocompromised individuals: a systematic review and meta-analysis using the GRADE framework
Source: Front Immunol. 2023 Sep 12;14:1204831. doi: 10.3389/fimmu.2023.1204831 (PMC10523015; doi:10.3389/fimmu.2023.1204831)
Supplement: Supplementary file 3 [file Table_3.docx]

Supplementary Material

Comparative Effectiveness of mRNA-1273 and BNT162b2 COVID-19 Vaccines in Immunocompromised Individuals: A Systematic Review and Meta-Analysis Using the GRADE Framework

**Xuan Wang, MD, MSc, Katrin Haeussler, PhD, MSc, Anne Spellman, PhD, MSc, Leslie E. Phillips, PhD, SM, Allison Ramiller, MPH, Mary T. Bausch-Jurken, PhD, MS, Pawana Sharma, MSc, Anna Krivelyova, MA, Sonam Vats, MPH, Nicolas Van de Velde, PhD***

*** Correspondence:** Corresponding Author: [Nicolas.VandeVelde@modernatx.com](mailto:Nicolas.VandeVelde@modernatx.com)

## Table S3. ROB Assessment per the NOS Scale for Cohort and Case-Control Studies

| **Cohort Studies** | | | | | | | | | |
| --- | --- | --- | --- | --- | --- | --- | --- | --- | --- |
| **Author, Year** | **Total Score** | **Representative-ness of Exposed Cohort^a^** | **Selection of Nonexposed Cohort^b^** | **Ascertain-ment of Exposure^c^** | **Out-come Not Present At Base-line^d^** | **Compar-ability of Cohorts^e^** | **Assess-ment of Outcome^f^** | **Sufficient Follow-Up Duration^g^** | **Ade-quate Follow-Up^h^** |
| Aslam, 2021 (1) | 7 stars | 1 star | 1 star | 1 star | 1 star | 0 star | 1 star | 1 star | 1 star |
| Embi,  2021 (2) | 7 stars | 1 star | 1 star | 1 star | 1 star | 2 stars | 1 star | 0 star | 0 star |
| Holroyd, 2022 (3) | 7 stars | 0 star | 1 star | 1 star | 1 star | 1 star | 1 star | 1 star | 1 star |
| Kelly,  2022 (4) | 8 stars | 1 star | 1 star | 1 star | 1 star | 1 star | 1 star | 1 star | 1 star |
| Khan, 2021 (5) | 8 stars | 1 star | 1 star | 1 star | 1 star | 1 star | 1 star | 1 star | 1 star |
| Liew,  2022 (6) | 5 stars | 1 star | 0 star | 1 star | 0 star | 0 star | 1 star | 1 star | 1 star |
| Malinis, 2021 (7) | 6 stars | 1 star | 0 star | 1 star | 1 star | 0 star | 1 star | 1 star | 1 star |
| Mazuecos, 2022 (8) | 8 stars | 1 star | 1 star | 1 star | 1 star | 1 star | 1 star | 1 star | 1 star |
| Mues, 2022 (9) | 9 stars | 1 star | 1 star | 1 star | 1 star | 2 star | 1 star | 1 star | 1 star |
| Patel,  2022 (10) | 8 stars | 1 star | 1 star | 1 star | 1 star | 1 star | 1 star | 1 star | 1 star |
| Piñana, 2022 (11) | 7 stars | 1 star | 1 star | 1 star | 1 star | 0 star | 1 star | 1 star | 1 star |
| Pino,  2022 (12) | 8 stars | 1 star | 1 star | 1 star | 1 star | 1 star | 1 star | 1 star | 1 star |
| Rooney, 2022 (13) | 6 stars | 1 star | 1 star | 0 star | 1 star | 0 star | 1 star | 1 star | 1 star |
| Sibbel, 2021 (14) | 8 stars | 1 star | 1 star | 1 star | 1 star | 1 star | 1 star | 1 star | 1 star |
| Yeo,  2022 (15) | 8 stars | 0 star | 1 star | 1 star | 1 star | 2 star | 1 star | 1 star | 1 star |
| Yetmar, 2022 (16) | 3 stars | 0 star | 0 star | 1 star | 0 star | 0 star | 1 star | 1 star | 0 star |
| **Case-Control Studies** | | | | | | | | | |
|  | **Total Score** | **Adequate Case Definition^i^** | **Represent-ativeness of Cases^j^** | **Selection of Controls^k^** | **Defin-ition of Controls^l^** | **Com-parability^m^** | **Ascer-tainment of Exposure^c^** | **Same Ascer-tainment Method For Cases^n^** | **Non-res-ponse Rate^o^** |
| Butt,  2022 (17) | 7 stars | 1 star | 1 star | 1 star | 1 star | 1 star | 1 star | 1 star | 0 star |

IC, immunocompromised; NOS, Newcastle-Ottawa Scale, RoB, risk of bias.

^a^1 star was awarded if the study population was truly or somewhat representative of a community or population. No star was given if the study population was sampled from a special population (eg, hospitalized patients).
^b^1 star was given if the nonexposed cohort (ie, non-IC cohort) was drawn from the same population as the exposed cohort (ie, IC cohort). If only 1 cohort of patients was included, no star was awarded.

^c^1 star was awarded if secured medical records or a structured interview was used to ascertain the IC condition. No star was awarded if the IC condition was self-reported or not described.

^d^1 star was given if the outcomes were assessed at the beginning of the study. No star was given if outcomes were not assessed at the beginning of the study.

^e^2 stars were given if the study was adjusted for the most important factors deliberately. 1 star was given if the study was adjusted for other important factors. If no adjustment was performed or there was no description of comparability, no star was awarded.

^f^1 star was given if the outcome was assessed from medical records or record linkage. No star was given if the outcome was self-reported.

^g^1 star was given if the duration of follow-up was >1 month, otherwise, no star was awarded.

^h^1 star was given if there was complete follow-up or the lost to follow-up rate was ≤20%. No star was awarded if the follow-up rate was <80% or if the follow-up rate was not reported.

^i^1 star was awarded if the case definition was adequate and independently validated. No star was given if the case definition was based on record linkage or self-reported, or not described.

^j^1 star was awarded if the cases were consecutive or a representative series of cases. No star was given if there was a potential for selection biases or the representativeness of cases was not stated.

^k^1 star was given if community controls were used. No star was awarded if hospital controls were used or controls were not described.

^l^1 star was given if the control had no history of disease. No star was given if no description was provided.

^m^2 stars were awarded if the study controlled for the most important factor as well as any additional factors.

^n^1 star was awarded if the method was the same. If a different method was used to ascertain cases and controls, no star was given.

^o^1 star was given if the nonresponse rate was the same for both cases and controls. No star was given if the nonresponse rate was different or not described.

**REFERENCES**

1. Aslam S, Adler E, Mekeel K, Little SJ. Clinical effectiveness of COVID-19 vaccination in solid organ transplant recipients. Transpl. Infect. Dis. (2021) 23:e13705. doi: 10.1111/tid.13705.

2. Embi PJ, Levy ME, Naleway AL, Patel P, Gaglani M, Natarajan K, et al. Effectiveness of 2-dose vaccination with mRNA COVID-19 vaccines against COVID-19–associated hospitalizations among immunocompromised adults—nine states, January–September 2021. MMWR Morb. Mortal. Wkly. Rep. (2021) 70:1553. doi: 10.15585/mmwr.mm7044e3.

3. Holroyd KB, Healy BC, Conway S, Houtchens M, Bakshi R, Bhattacharyya S, et al. Humoral response to COVID-19 vaccination in MS patients on disease modifying therapy: immune profiles and clinical outcomes. Mult Scler Relat Disord (2022) 67:104079. doi: 10.1016/j.msard.2022.104079.

4. Kelly JD, Leonard S, Hoggatt KJ, Boscardin WJ, Lum EN, Moss-Vazquez TA, et al. Incidence of severe COVID-19 illness following vaccination and booster with BNT162b2, mRNA-1273, and Ad26.COV2.S vaccines. JAMA (2022) 328:1427-1437. doi: 10.1001/jama.2022.17985.

5. Khan N, Mahmud N. Effectiveness of SARS-CoV-2 vaccination in a veterans affairs cohort of patients with inflammatory bowel disease with diverse exposure to immunosuppressive medications. Gastroenterology (2021) 161:827-836. doi: 10.1053/j.gastro.2021.05.044.

6. Liew J, Gianfrancesco M, Harrison C, Izadi Z, Rush S, Lawson-Tovey S, et al. SARS-CoV-2 breakthrough infections among vaccinated individuals with rheumatic disease: results from the COVID-19 Global Rheumatology Alliance provider registry. RMD Open (2022) 8:e002187. doi: 10.1136/rmdopen-2021-002187.

7. Malinis M, Cohen E, Azar MM. Effectiveness of SARS-CoV-2 vaccination in fully vaccinated solid organ transplant recipients. Am. J. Transplant. (2021) 21:2916-2918. doi: 10.1111/ajt.16713.

8. Mazuecos A, Villanego F, Zarraga S, López V, Oppenheimer F, Llinàs-Mallol L, et al. Breakthrough infections following mRNA SARS-CoV-2 vaccination in kidney transplant recipients. Transplantation (2022) 106:1430-1439. doi: 10.1097/tp.0000000000004119.

9. Mues KE, Kirk B, Patel DA, Gelman A, Chavers S, Talarico C, et al. Real-world comparative effectiveness of mRNA-1273 and BNT162b2 vaccines among immunocompromised adults identified in adminstrative claims data in the United States. Vaccine (2022) 40:6730-6739. doi: 10.1101/2022.05.13.22274960.

10. Patel NJ, Wang X, Fu X, Kawano Y, Cook C, Vanni KMM, et al. Factors associated with COVID-19 breakthrough infection among vaccinated patients with rheumatic diseases: a cohort study. Semin. Arthritis Rheum. (2022) 58:152108. doi: 10.1016/j.semarthrit.2022.152108.

11. Pinana JL, Lopez-Corral L, Martino R, Vazquez L, Perez A, Martin-Martin G, et al. SARS-CoV-2 vaccine response and rate of breakthrough infection in patients with hematological disorders. J. Hematol. Oncol. (2022) 15:54. doi: 10.1186/s13045-022-01275-7.

12. Pino MS, Cheli S, Perna M, Fabbroni V, Giordano C, Martella F, et al. The national COVID-19 vaccination campaign targeting the extremely vulnerable: the Florence Medical Oncology Unit experience in patients with cancer. Eur. J. Cancer (2022) 170:149-157. doi: 10.1016/j.ejca.2022.04.008.

13. Rooney A, Bivona C, Liu B, Streeter D, Gong H, Khan Q. Risk of SARS-CoV-2 breakthrough infection in vaccinated cancer patients: a retrospective cohort study. J. Hematol. Oncol. (2022) 15:67. doi: 10.1186/s13045-022-01290-8.

14. Sibbel S, McKeon K, Luo J, Wendt K, Walker AG, Kelley T, et al. Real-world effectiveness and immunogenicity of BNT162b2 and mRNA-1273 SARS-CoV-2 vaccines in patients on hemodialysis. J. Am. Soc. Nephrol. (2022) 33:49-57. doi: 10.1681/ASN.2021060778.

15. Yeo T, Quek AML, Yong KP, Tye JSN, Ratnagopal P, Soon DTL, et al. COVID-19 infection after two doses of SARS-CoV-2 mRNA vaccine in multiple sclerosis, AQP4-antibody NMOSD and MOGAD. Mult Scler Relat Disord (2022) 65:104003. doi: 10.1016/j.msard.2022.104003.

16. Yetmar ZA, Bhaimia E, Bierle DM, Ganesh R, Razonable RR. Breakthrough COVID-19 after SARS-CoV-2 vaccination in solid organ transplant recipients: an analysis of symptomatic cases and monoclonal antibody therapy. Transpl. Infect. Dis. (2022) 24:e13779. doi: 10.1111/tid.13779.

17. Butt AA, Talisa VB, Yan P, Shaikh OS, Omer SB, Mayr FB. Real-world effectiveness of the severe acute respiratory syndrome coronavirus 2 (SARS-CoV-2) mRNA vaccines in preventing confirmed infection in patients on chronic hemodialysis. Clin. Infect. Dis. (2022) 75:e617-e622. doi: 10.1093/cid/ciac118.
